# Abiotic Deposition of Fe Complexes onto *Leptothrix* Sheaths

**DOI:** 10.3390/biology5020026

**Published:** 2016-06-03

**Authors:** Tatsuki Kunoh, Hideki Hashimoto, Ian R. McFarlane, Naoaki Hayashi, Tomoko Suzuki, Eisuke Taketa, Katsunori Tamura, Mikio Takano, Mohamed Y. El-Naggar, Hitoshi Kunoh, Jun Takada

**Affiliations:** 1 Core Research for Evolutionary Science and Technology (CREST), Japan Science and Technology Agency (JST), Okayama 700-0082, Japan; tkunoh06@cc.okayama-u.ac.jp (T.K.); hideki-h@cc.kogakuin.ac.jp (H.H); hayashi@icems.kyoto-u.ac.jp (N.H.); suzukit@fc.jwu.ac.jp (T.S.); en421232@s.okayama-u.ac.jp (E.T.); ktamura@okayama-u.ac.jp (K.T.); takano@cc.okayama-u.ac.jp (M.T.); hkunoh@cc.okayama-u.ac.jp (H.K.); 2 Graduate School of Natural Science and Technology, Okayama University, Okayama 700-0082, Japan; 3 Department of Applied Chemistry, School of Advanced Engineering, Kogakuin University, Hachiohji, Tokyo 192-0015, Japan; 4 Department of Physics and Astronomy, University of Southern California, Los Angeles, CA 90089, USA; ian.r.mcfarlane@gmail.com; 5 Department of Chemical and Biological Science, Japan Woman’s University, Bunkyo-ku, Tokyo 112-8681, Japan; 6 Molecular and Computational Biology Section, Department of Biological Sciences, University of Southern California, Los Angeles, CA 90089, USA; 7 Department of Chemistry, University of Southern California, Los Angeles, CA 90089, USA

**Keywords:** *Leptothrix cholodnii* SP-6, abiotic oxidation, Fe(III) particles, sheath, direct deposition

## Abstract

Bacteria classified in species of the genus *Leptothrix* produce extracellular, microtubular, Fe-encrusted sheaths. The encrustation has been previously linked to bacterial Fe oxidases, which oxidize Fe(II) to Fe(III) and/or active groups of bacterial exopolymers within sheaths to attract and bind aqueous-phase inorganics. When *L. cholodnii* SP-6 cells were cultured in media amended with high Fe(II) concentrations, Fe(III) precipitates visibly formed immediately after addition of Fe(II) to the medium, suggesting prompt abiotic oxidation of Fe(II) to Fe(III). Intriguingly, these precipitates were deposited onto the sheath surface of bacterial cells as the population was actively growing. When Fe(III) was added to the medium, similar precipitates formed in the medium first and were abiotically deposited onto the sheath surfaces. The precipitates in the Fe(II) medium were composed of assemblies of globular, amorphous particles (*ca.* 50 nm diameter), while those in the Fe(III) medium were composed of large, aggregated particles (≥3 µm diameter) with a similar amorphous structure. These precipitates also adhered to cell-free sheaths. We thus concluded that direct abiotic deposition of Fe complexes onto the sheath surface occurs independently of cellular activity in liquid media containing Fe salts, although it remains unclear how this deposition is associated with the previously proposed mechanisms (oxidation enzyme- and/or active group of organic components-involved) of Fe encrustation of the *Leptothrix* sheaths.

## 1. Introduction

The Fe/Mn-oxidizing bacteria such as *Leptothrix* and *Gallionella* species are ubiquitous habitants in aqueous environments, especially at groundwater outwelling sites which are characterized by a nearly neutral pH, an oxygen gradient, and a source of reduced Fe and Mn minerals [1,2]. The *Leptothrix* species have the potential to produce extracellular, microtubular sheaths with the precipitation of copious amounts of oxidized Fe or Mn [1,2]. When actively multiplying, *Leptothrix* cells divide to form catenulate cells and secrete exopolymers from their surface, which provide a platform for the formation of the sheaths enriched in metals, Fe in particular [3,4]. Seemingly sturdy, yellowish brown sheaths are formed by binding these bacterial organic secretions to aqueous-phase inorganics such as Fe, Si, P, and often Ca [4,5,6,7,8]. Enzymatic reactions have been proposed to play a role, and Fe-/Mn-oxidizing proteins were identified and shown to be excreted from bacterial cells in the spent culture medium of *L. discophora* SS-1 [9]. In addition, metal-oxidizing enzymes have been suggested to play a role in the formation and metal encrustation of the *Leptothrix* sheath [10,11,12,13]. Therefore, encrustation of inorganics in sheaths is arguably a result of biotic metal oxidation, and the associated reactions may even drive the chemolithoautotrophic energy metabolism of *L. ochracea* [14]. In spite of this background knowledge, the precise mechanism of the interactions between bacterial organics and aqueous-phase inorganics for sheath formation has continued to be a matter of debate.

Ferris *et al.* [15] reported that the metallic ions in natural bodies of water were very often influenced by specific aqueous-phase inorganics and biogenic organic materials, suggesting complicated interactions among the various metal-complexing agents in aquatic systems and microorganisms and their constituent polymers. Microbiologically produced Fe-complexing ligands have thus been hypothesized to play critical roles in the delivery of Fe(II) to Fe(II) or Fe(III) hydroxide/oxyhydroxide and in the limited crystallinity of Fe(III) oxyhydoxides observed within bacterial biofilms [16]. Such complex interactions must occur during incubation of *Leptothrix* in the Fe-containing media that contain various inorganic and organic components.

Multiple researchers have cultured isolated strains of *Leptothrix* in media with various Fe sources such as FeCl_2_, FeSO_4_, ferric ammonium citrate, FeCl_3_, Fe plate, and Fe powder [17,18,19,20,21] for understanding the mechanism of the Fe oxidation and deposition on sheaths. Since abiotic Fe oxidation in fully oxygenated water at circumneutral pH is very rapid (half-life < 1 min), this instantaneous precipitation of Fe oxyhydroxides could potentially encase a cell in a metal oxide cluster [14,22]. Because Fe ions form hydroxide/oxyhydroxide complexes and diverse salts with other elements [23], understanding the mode and behavior of abiotic oxidation products in Fe-, particularly Fe salt-containing media, will be a valuable aid in precisely assessing the kinetics of biotic iron oxidation in proximity of microbial cell surfaces and their associated structures such as sheaths, as was emphasized previously [18].

Here we provide microscopic and spectroscopic evidence that Fe(III) precipitates are first generated from Fe(II) by abiotic oxidation in the medium and then are deposited onto sheaths of cultured *Leptothrix* cells directly while maintaining their morphology, crystallinity, and inorganic components.

## 2. Materials and Methods

### 2.1. Strains, Medium, and Culturing

*Leptothrix cholodnii* strain SP-6 (ATCC 51168) were transferred from frozen stock onto MSVP agar [24] with sterile toothpicks and incubated at 20 °C for several days. Single colonies were then independently transferred to 25 mL of MSVP broth with sterile toothpicks and incubated on a rotary shaker (EYELA FMC-1000, Tokyo Rikakikai, Tokyo, Japan) at 20 °C and 70 rpm. After 2–3 days, 1 mL of the cell suspension (adjusted to 10 cfu/mL by densitometry using a NanoDrop 2000C spectrophotometer, Thermo Fisher Scientific, Waltham, MA, USA) was transferred to 100 mL of MSVP in glass flasks, and incubated as above. For media with various amounts of Fe source, Fe-lacking MSVP (MSVP-FeSO_4_) was supplemented with 10–500 µM FeSO_4_ or FeCl_2_, and 5–250 µM Fe_2_(SO_4_)_3_.

### 2.2. Colony-Forming Unit (cfu) Test to Examine Growth of Cell Population

Following a previous procedure [25], exponentially growing cells cultured in 100 mL of the respective media were harvested on designated days after inoculation, then serially diluted 10-fold with MSVP. These dilutions were immediately spread on MSVP agar plates. After a four-day incubation at 20 °C, the colonies for three replicates were counted to determine mean cfu/mL. Because this bacterium forms chains and flocs while it multiplies, the present cfu/mL values are only rough estimates of the bacteria population.

### 2.3. Lysozyme-EDTA-SDS Bacteriolysis

Following a previous procedure [12], after three days in MSVP, clusters of SP-6 colonies and the accompanying sheaths were harvested by centrifugation at 3,600 × *g* for 10 min. The pellet was washed in ultrapure water (UPW) and suspended in 3 mL of the lysis solution containing 2.5 mM EDTA and 150 µg of lysozyme (Sigma-Aldrich, St. Louis, MO, USA) per mL. The reaction mixture was incubated for 30 min at 37 °C. Then 10% SDS was added at a final concentration of 1%, and the resultant mixture was incubated for 30 min at room temperature in a shaking mixer (LMS Co. Ltd., Tokyo, Japan). The specimens, collected by centrifugation, were washed in sterile UPW and then MSVP-Fe at least five times. Successful digestion of the cells was confirmed by phase contrast microscopy (BX51 System Microscope, OLYMPUS, Tokyo, Japan). The final cell-free sheaths were incubated in MSVP-Fe supplemented with varied concentrations of FeSO_4_, FeCl_2_, or Fe_2_(SO_4_)_3_ for two days to check for abiotic Fe deposition onto the sheaths.

### 2.4. Determination of Fe(II) and Total Fe Concentration in Culture Medium with O-Phenanthroline

The time course of concentration changes in Fe(II) and total Fe in MSVP-Fe supplemented with various concentrations of Fe sources was determined during incubation using a published method [26] with modifications. The respective medium (2.5 mL), sampled at the designated times after the onset of incubation, was added to 0.25 mL of acetic acid buffer (pH 4.6) and 5 mM *O*-phenanthroline, which was then brought up to 5 mL with UPW. After gentle agitation, the reaction mixture stood at room temperature for 30 min to yield the reddish orange Fe(II)-chelate with *O*-phenanthroline, followed by determination of absorbance (OD) at 510 nm. The background absorbance at 700 nm was subtracted from the measured value at 510 nm with the NanoDrop 2000C spectrophotometer (Thermo Fisher Scientific). To determine the total Fe concentration, 0.5 mL of 3 N HCl was added to 2.5 mL of the sampled medium and heated in boiling water for 5 min. To reduce acid-soluble Fe(III), 0.1 mL of 1.44 M HCl-hydroxyamine was added to the reaction mixture, followed by 0.25 mL of 5 mM *O*-phenanthroline. After the pH was adjusted to 3.5 with 6 N aqua ammonia, 0.25 mL of acetic acid buffer (pH 4.6) was added to the reaction mixture. The total volume was then brought up to 5 mL with UPW and, after 30 min, absorbance at 510 nm was measured. Theoretically, the acid and hydroxylamine treatments should reduce Fe(III) to Fe(II), which has a maximal absorbance at 510 nm. Thus, the total Fe concentration is technically equivalent to the concentration of acid-soluble Fe in the medium. It is notable that Fe(III)-chelate with *O*-phenanthroline does not absorb 510 nm wavelengths. Total Fe concentration was estimated using a standard curve based on serial dilutions of 1 g/L ammonium Fe(II) sulfate in 0.012 N HCl.

### 2.5. Scanning Electron Microscopy (SEM) and Energy Dispersive X-Ray Spectroscopy (EDX)

Collected sheaths were fixed overnight with 2.5% glutaraldehyde (Sigma-Aldrich) at 4 °C and then dehydrated in a graded ethanol series (10, 25, 50, 75, 90, 100%) at 10-min intervals. At each dehydration step, specimens were collected by centrifugation at 2,400 × *g* for 5 min. When necessary, the specimens were fixed further with 1% OsO_4_ in 0.1 M cacodylate buffer (pH 7.0) for 15 min, then washed three times with distilled water before the ethanol dehydration. The specimens were air-dried on a 0.22 µm-pore size filter (Whatman, GE Healthcare, Pittsburgh, PA, USA) or freeze-dried after solvent exchange from ethanol to *t*-butyl alcohol and then stuck to carbon tape (Nisshin EM, Tokyo, Japan) on an SEM stub. The SEM imaging and component analyses were performed with a JSM-7001F, JSM-6610 (JEOL, Tokyo, Japan) (at USC), or S-4300 equipped with an energy dispersive X-ray spectrometer (EDX) (Hitachi, Tokyo, Japan) (at OU) at an accelerating voltage of 15 kV. For SEM imaging without component analyses by EDX, the specimens were coated with platinum. Atomic percentages of major inorganics (Fe, S, P, Na, Ca, and Mg) detectable in sheaths were measured by EDX, and variation among 10 spot values was calculated. Atomic percentages of Fe described above were based on a total percentage of these six elements.

### 2.6. Transmission Electron Microscopy (TEM) Imaging and EDX Elemental Mapping

Precipitates were harvested from inoculated and uninoculated MSVP-Fe containing 300 µM FeSO_4_, 300 µM FeCl_2_, or 150 µM Fe_2_(SO_4_)_3_ by centrifugation at 2,400 × *g* for 10 min and then washed once in ethanol. A few drops of the resultant suspensions were placed on carbon-coated copper grids (Nisshin EM) and air-dried for imaging with TEM, high angle annular dark-field (HAADF), secondary electron (SE) and EDX elemental mapping using a JEOL JEM-2100F TEM equipped with a CEOS Cs corrector (for spherical correction) and an EDX detector (JED-2300T; JEOL), respectively.

## 3. Results

### 3.1. Influence of Fe(II) Concentration in Medium on Growth of SP-6 Cells

**Figure 1:**
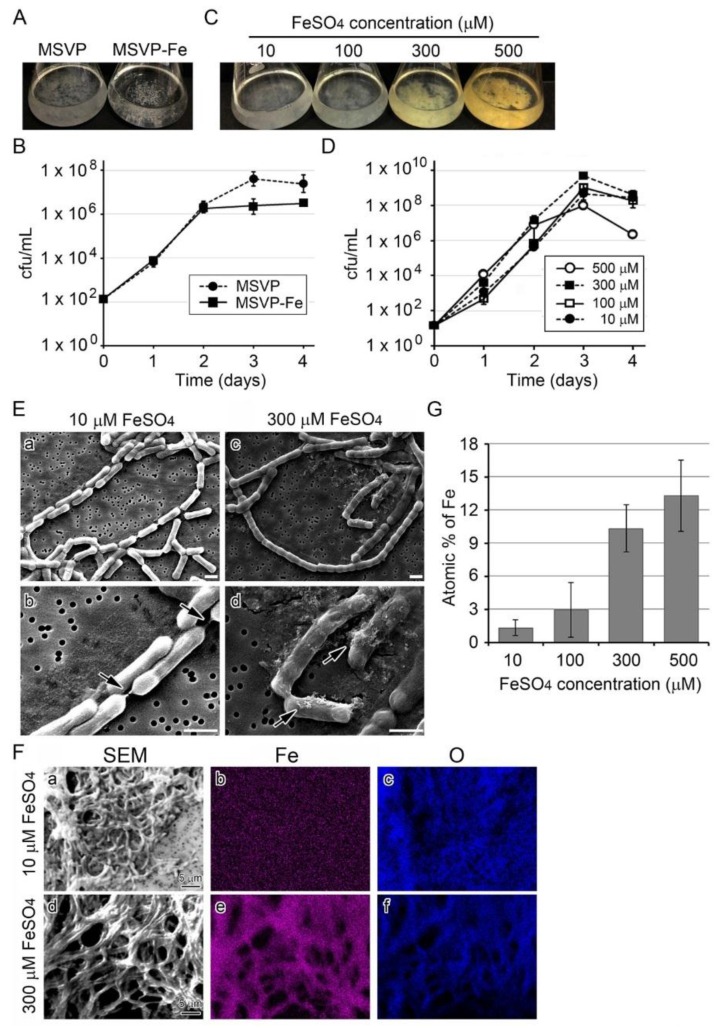
Growth of SP-6 cells and their sheath formation in media containing different amounts of FeSO_4._ (**A**) Fluffy colony clusters of SP-6 in MSVP medium (left) and aggregated, granular colony clusters in MSVP-Fe medium (right) after a three-day incubation. (**B**) Time course of cell growth (cfu/mL) in MSVP and MSVP-Fe media. Expressed as mean ± s. d. for six replicates. (**C**) Fluffy colony clusters in 10 to 500 µM FeSO_4_ media after a three-day incubation. Note that the color of the clusters differs: whitish in 10 and 100 µM FeSO_4_ media and brownish in 300 and 500 µM FeSO_4_ media. (**D**) Time course of cell growth (cfu/mL) in media containing different amounts of FeSO_4._ Note that cell growth in 500 µM FeSO_4_ medium declined rapidly compared with that in other media. (**E**) SEM images of sheaths formed in 10 (**a**,**b**) or 300 µM (**c**,**d**) FeSO_4_ medium after a three-day incubation. Note the thin sheath enveloping the catenulate cells in 10 µM FeSO_4_ medium and seemingly sturdy sheath in 300 µM FeSO_4_ medium. Arrows show shrunken sheaths at the cell junctions in (**b**) and precipitates adhering on sheath surface in (**d**), respectively. Scale bars = 1 µm. (**F**) EDX mapping of Fe (**b**,**e**) and O (**c**,**f**) in sheaths harvested from 10 µM (**a**–**c**) or 300 µM (**d**–**f**) FeSO_4_ medium after a three-day incubation. (**G**) Mean atomic percentage (± s. d.) of Fe in sheaths harvested from 10 to 500 µM FeSO_4_ media after a three-day incubation (measured by EDX). Expressed as mean ± s. d. from *N* = 10 spots per sample.

The MSVP medium currently used for culturing SP-6 contains 10 µM FeSO_4_ [24]. When SP-6 is cultured in MSVP for three days, whitish clusters of fluffy colonies formed on the bottom and wall of the flasks, but in MSVP-Fe (MSVP lacking FeSO_4_) aggregated colonies formed only on the flask bottom (Figure 1A). The subsequent test to determine the number of colony forming units (hereafter, the cfu test) (Figure 1B) showed that cell growth increased exponentially in MSVP through day 3 before the start of the stationary phase, but cell growth in MSVP-Fe had already reached the stationary phase by day 2. These results showed that SP-6 can grow in the Fe-lacking medium to some extent, but Fe(II) might be necessary for cells to continue to multiply. Although the clusters in MSVP-Fe looked visibly smaller than in MSVP (Figure 1A), light and scanning electron micrographs (SEM) showed similar thin, nearly transparent sheaths around chained cells in the clusters in both media by day 3 (Figure S1 A,B), suggesting that the presence of Fe(II) might not be essential for the formation of at least the initial sheath frame.

The growth requirement of cells for Fe(II) was evaluated in more detail by culturing SP-6 cells in MSVP-Fe supplemented with varied concentrations of FeSO_4_ (10–500 µM) (hereafter, simply referred to as 10–500 µM FeSO_4_ medium). Similar clusters of fluffy colony formed in all media, but the color of the clusters changed from whitish (≤100 µM FeSO_4_) to yellowish brown (≥300 µM FeSO_4_) within three days, which probably reflects the progression of Fe encrustation in sheaths with increased Fe(II) concentrations in the medium (Figure 1C). The cfu test indicated that cell growth increased exponentially in all media through day 3 and tended to decline thereafter, regardless of Fe concentration in media. Growth in 500 µM FeSO_4_ medium declined significantly rapidly (*p* < 0.05) compared with that in the presence of ≤300 µM FeSO_4_ (Figure 1D). Taken together, these results suggest that (i) the cells can form the initial sheath frame regardless of the presence or absence of Fe(II) in the medium, (ii) progression of Fe-encrustation in sheaths largely depends on the Fe(II) concentration in the medium, and (iii) 500 µM FeSO_4_ might have some adverse but not lethal effects on cell growth.

### 3.2. Influence of Fe(II) Concentration on Fe Deposition onto Sheaths

Since the colony clusters in a 300 or 500 µM FeSO_4_ medium looked brownish and those in 10 or 100 µM FeSO_4_ media were whitish (Figure 1C), we assumed that the color difference could be ascribed to the degree of Fe deposition onto sheaths. To verify this assumption, we used scanning electron microscopy (SEM) and energy dispersive x-ray spectroscopy (EDX) to compare the sheaths formed in 10 µM FeSO_4_ medium with those in 300 µM FeSO_4_ medium. In 10 µM FeSO_4_ medium, thin, smooth sheaths encompassed the catenulate cells whose junctions were connected with shrunken, thread-like sheaths (Figure 1E a,b), while in 300 µM FeSO_4_ medium, the catenulate cells were covered entirely with seemingly sturdy, thick sheaths (Figure 1E c,d). It was notable that deposits aggregated on the latter sheaths (Figure 1E d, arrows). In contrast, such deposits were not seen near or on the smooth surface of the former sheaths. We considered that such deposits could be Fe precipitates formed in the medium. The EDX analysis showed Fe in sheaths from the 300 µM FeSO_4_ medium, but none was detectable in sheaths from the 10 µM FeSO_4_ medium, although O was detected in both sheaths (Figure 1F). As expected, the Fe atomic percentage in sheaths of the 10 µM FeSO_4_ medium was quite low (*ca.* 1%), but it increased with increasing concentrations of FeSO_4_ to *ca.* 17% in 300 µM FeSO_4_ medium (Figure 1G). These results show that Fe deposition onto sheaths is apparently enhanced as Fe(II) concentration increases in the medium, consistent with an earlier description [8].

### 3.3. Deposition of Fe-Containing Precipitates onto Sheaths in Media Containing Higher Concentrations of the Fe Source

**Figure 2:**
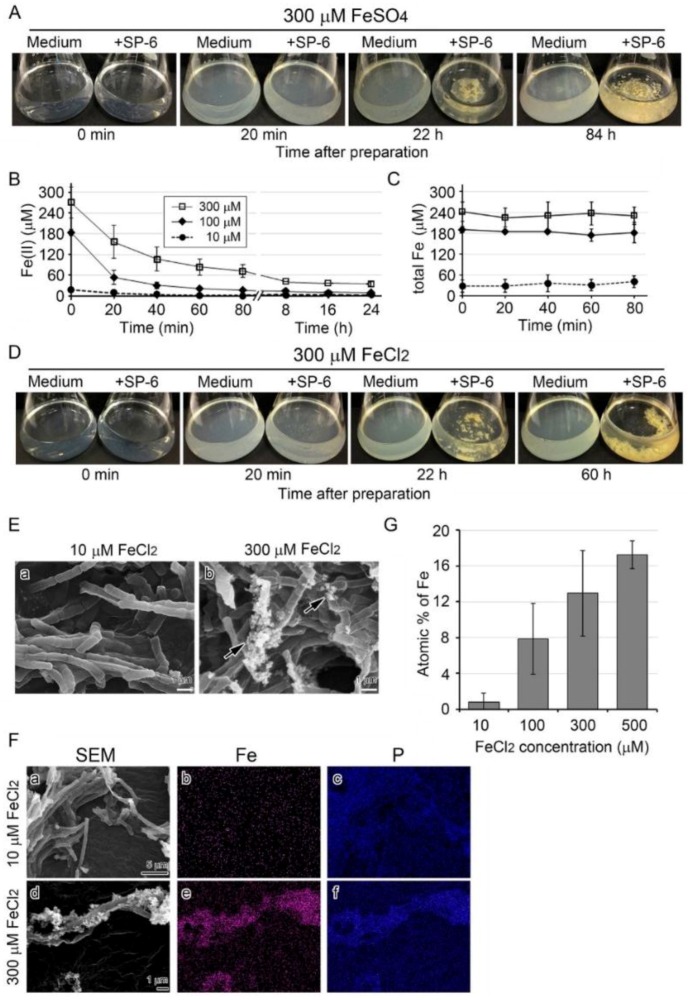
Growth of SP-6 cells and their sheath formation in media containing FeSO_4_ or FeCl_2_ and occurrence of abiotic precipitation. (**A**) Visual changes in medium turbidity and colony formation in inoculated and uninoculated 300 µM FeSO_4_ media. Note that the medium became turbid within 20 min after preparation, but the turbidity in the inoculated medium cleared as bacterial cells multiplied. (**B**,**C**) Time course of change in mean concentration of Fe(II) and total Fe in media containing different amounts of FeSO_4_. Expressed as mean ± s. d. from at least three replicates. (**D**) Visual changes in medium turbidity and colony formation in inoculated and uninoculated 300 µM FeCl_2_ media. Note that the visual turbidity change was quite similar to that of the FeSO_4_ medium. (**E**) SEM images of sheaths harvested from 10 or 300 µM FeCl_2_ medium after a three-day incubation. Note the sheath with a smooth surface in 10 µM FeCl_2_ medium (**a**) and the sheath with precipitates (arrows) adhering to its surface in 300 µM FeCl_2_ medium (**b**). (**F**) EDX mapping of Fe (**b**,**e**) or P (**c**,**f**) in sheaths harvested from 10 µM (**a**–**c**) or 300 µM (**c**–**f**) FeCl_2_ medium after a three-day incubation. Note the heavy deposition of Fe and P in precipitates adhering to 300 µM FeCl_2_ medium. (**G**) Atomic percentages of Fe in sheaths harvested from 10 to 500 µM FeCl_2_ media after a three-day incubation (determined by EDX). Expressed as mean ± s. d. from *N*= 10 spots per sample.

The MSVP-Fe medium became visually turbid within 20 min after addition of 10 to 300 µM FeSO_4_, although it was clear immediately after it was prepared (Figure 2A). The turbidity was the most prominent with 300 or 500 µM FeSO_4_ from large amounts of precipitates. When bacterial cells were added to the 300 µM FeSO_4_ medium, the turbidity cleared by 22 h and remained clear thereafter. As expected, the uninoculated medium remained turbid for 84 h after preparation (*i.e.*, the end of the experiment) (Figure 2A).

The brownish colony clusters on the bottom of the inoculated flasks were considered to reflect deposition of the turbid material (plausibly Fe-hydroxides and/or Fe-oxyhydroxides formed by abiotic oxidation [14]) onto sheaths. The brownish color of the clusters may reflect the formation of different types of ferric oxide minerals with distinct crystal structures upon deposition onto sheaths.

The colorimetric iron determination using *O*-phenanthroline revealed that the Fe(II) concentration in 10–300 µM FeSO_4_ media began to decline within 20 min and was less than 60 µM within 24 h (Figure 2B), suggesting that the media had undergone rapid abiotic oxidation, as previously noted [14,22]. In contrast, the total Fe concentration remained nearly constant in all media for 80 min (Figure 2C). Similarly, in the 300 µM FeCl_2_ medium, the turbidity seen immediately after preparation cleared by 22 h with growth of inoculated cells (Figure 2D).

The Mössbauer analysis showed that precipitates collected from the uninoculated medium 12 h after preparation comprised *ca.* 99% of Fe(III) and 1% of Fe(II) (Figure S2). The SEM of sheaths in the 10 µM FeCl_2_ medium showed relatively smooth surfaces (Figure 2E a), whereas in the 300 µM FeCl_2_ medium, precipitates had aggregated on the sheath surfaces (Figure 2E b, arrows). Subsequent EDX mapping detected P but not Fe in sheaths in the 10 µM FeCl_2_ medium, but detected both P and Fe in sheaths and the aggregated precipitates in the 300 µM FeCl_2_ medium (Figure 2F). The average atomic percentages of Fe in 10 spots of sheaths harvested after three days were 0.8 ± 1.1, 7.9 ± 3.9, 13.0 ± 4.8, and 17.4 ± 1.6 % in 10, 100, 300, and 500 µM FeCl_2_ media, respectively, indicating that deposition of Fe precipitates onto the sheath surfaces increased as the Fe source increased in the medium (Figure 2G).

### 3.4. Recruitment of Precipitates in Media Containing Fe(III) for Fe Deposition onto Sheaths

**Figure 3:**
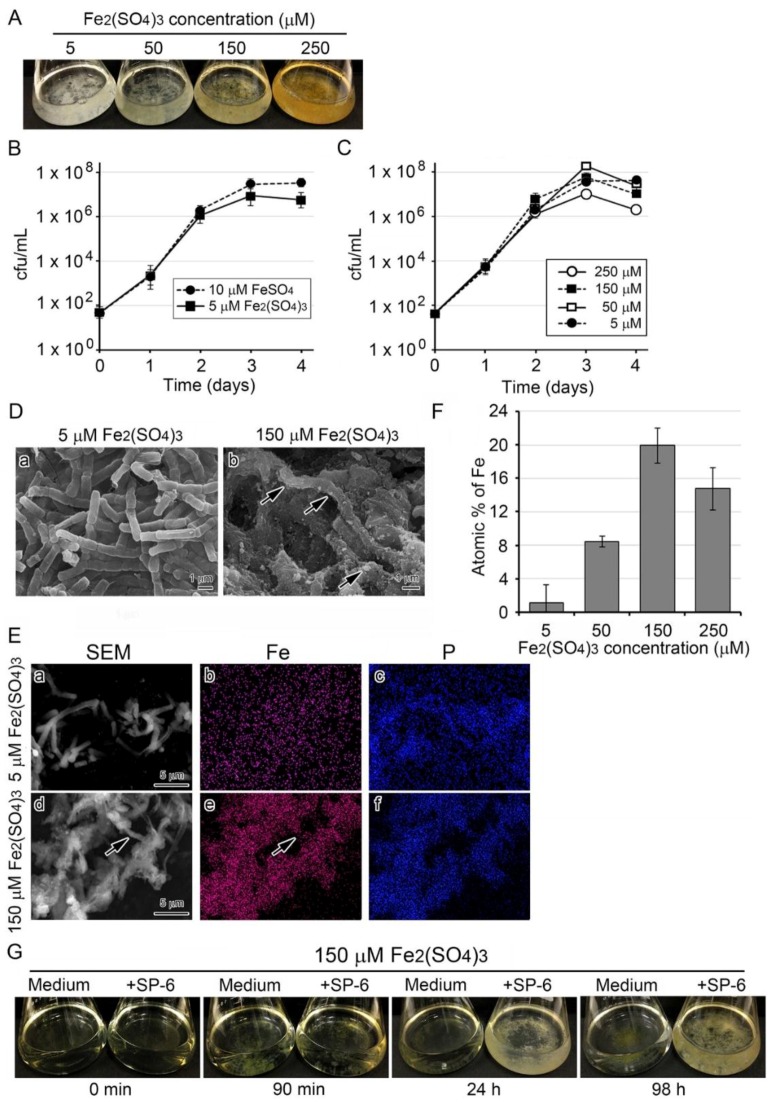
Growth of SP-6 cells and their sheath formation in media containing Fe_2_(SO_4_)_3_ and occurrence of abiotic precipitation. (**A**) Fluffy colony clusters formed in media containing 5 to 250 µM Fe_2_(SO_4_)_3_ by day 3 of incubation. Note the deeply colored medium and cluster in 250 µM Fe_2_(SO_4_)_3_ medium. (**B**) Time course of cell growth (cfu/mL) in 5 µM Fe_2_(SO_4_)_3_ medium compared with that in 10 µM FeSO_4_ medium. Expressed as mean ± s. d. from six replicates. (**C**) Time course of cell growth (cfu/mL) in 5 to 250 µM Fe_2_(SO_4_)_3_ media. (**D**) SEM images of sheaths harvested from inoculated 5 µM (**a**) and 300 µM Fe_2_(SO_4_)_3_ (**b**) media. Note the sheath with a smooth surface in the 5 µM Fe_2_(SO_4_)_3_ medium (**a**) and the sheath with precipitates (arrows) adhering to its surface in the 300 µM Fe_2_(SO_4_)_3_ medium (**b**). Scale bar = 1 µm. (**E**) EDX mapping of Fe (**b**,**e**) or P (**c**,**f**) in sheaths harvested from 5 µM (**a**–**c**) and 50 µM Fe_2_(SO_4_)_3_ (**d**–**f**) media after a three-day incubation. Note that Fe was not detected in the sheaths from 5 µM Fe_2_(SO_4_)_3_ in contrast to heavy deposition of Fe in the sheaths from 50 µM Fe_2_(SO_4_)_3_. (**F**) Atomic percentages of Fe on sheaths harvested from inoculated 5 to 250 µM Fe_2_(SO_4_)_3_ media (determined by EDX; expressed as mean ± s. d. from *N* = 10 spots per sample). (**G**) Visual changes in inoculated and uninoculated 150 µM Fe_2_(SO_4_)_3_ medium.

Like the FeSO_4_ and FeCl_2_ media, freshly prepared media supplemented with 5 to 250 µM Fe_2_(SO_4_)_3_ were transparent. Unlike FeSO_4_ or FeCl_2_ medium, however, instead of becoming turbid, they formed fluffy precipitates at the bottom of the flask within 1 h. The precipitates became more prominent and more intensely brown with increasing Fe levels in the medium. After bacterial cells were added to the media, colony clusters mingled with the existing precipitates, and the medium, although still transparent, turned slightly to deeply brownish, depending on the Fe concentrations by day 3 after inoculation (Figure 3A). The subsequent cfu test (Figure 3B) showed that cell growth increased almost exponentially through day 2 and reached the stationary phase by day 3 similarly in 10 µM FeSO_4_ and 5 µM Fe_2_(SO_4_)_3_ media. Exponential cell growth was similar to that in 5 to 250 µM Fe_2_(SO_4_)_3_ media through day 2 and tended to decline by day 4 after the stationary phase around day 3 (Figure 3C). The SEM images showed that in the 5 µM Fe_2_(SO_4_)_3_ medium, the chained cells were covered with thin, smooth sheaths (Figure 3D a), while in the 150 µM Fe_2_(SO_4_)_3_ medium, they were covered with rugged sheaths shouldering abundant precipitates (Figure 3D b, arrows).

The EDX mapping of sheaths harvested after a three-day incubation showed that in sheaths from the 5 µM Fe_2_(SO_4_)_3_ medium, P was detected but Fe was not, while in sheaths of 150 µM Fe_2_(SO_4_)_3_ medium both P and Fe were detected (Figure 3E, arrows). The atomic percentage of Fe in sheaths tended to be dependent on the Fe(III) concentration in the medium up to 150 µM (Figure 3F). However, it was lower in sheaths of the 250 µM Fe_2_(SO_4_)_3_ medium. Considering the standard deviation, the percentages in 150 and 250 µM Fe_2_(SO_4_)_3_ media do not substantially differ, suggesting that the deposition of Fe_2_(SO_4_)_3_ on the sheaths could reach a saturation level at 150 µM.

These results raised the assumption that the precipitation resulted from the conversion of Fe_2_(SO_4_)_3_ to Fe(III) hydroxides/oxyhydroxides [23] and that these precipitates can aggregate on the surface of initial sheaths in a 150 µM Fe_2_(SO_4_)_3_ medium. Also supporting this assumption, the fluffy Fe(III) precipitates that formed within 1.5 h after medium preparation disappeared from the medium and aggregated on the sheath surface, leading to the formation of relatively large clusters of dappled brownish colonies by 98 h (Figure 3G). The dappled brownish coloration is probably due to uneven adherence of the precipitates to the sheath surfaces, as shown (Figure 3D b, E d).

### 3.5. Deposition of Media-Formed Fe Precipitates onto Initial Sheaths

**Figure 4:**
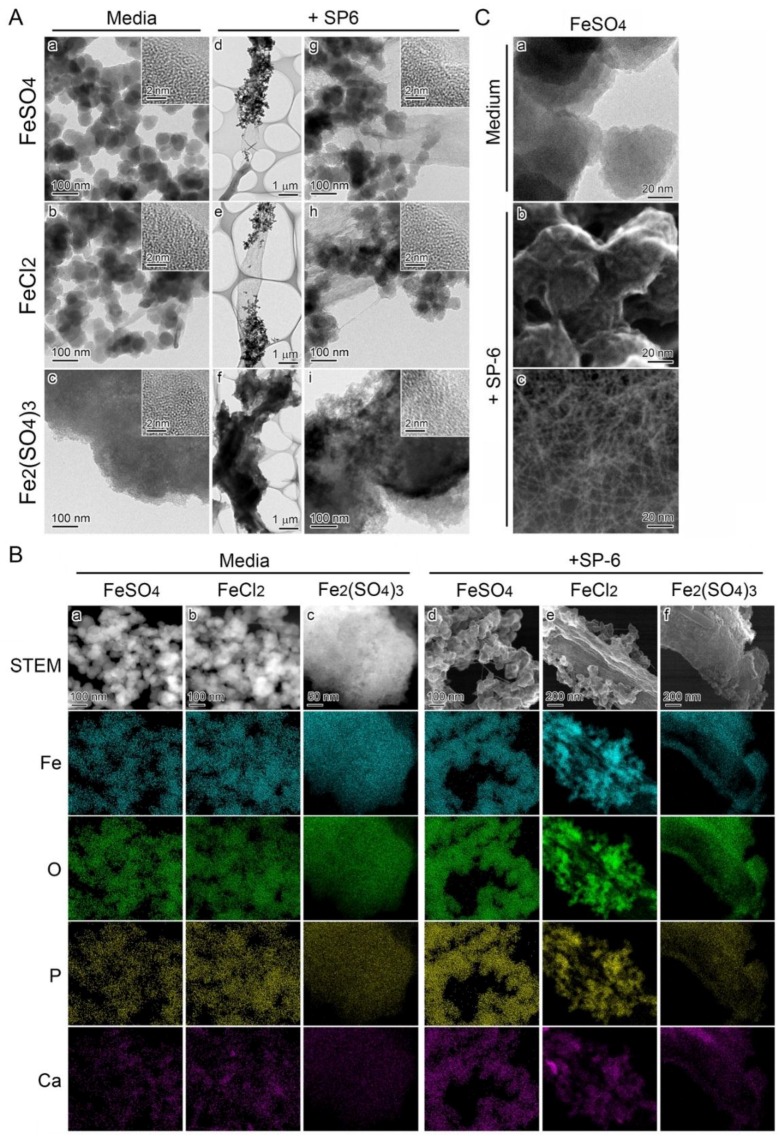
Morphology and inorganic composition of precipitates adhering to SP-6 sheaths. (**A**) STEM images of precipitates in uninoculated 300 µM FeSO_4_ (**a**), 300 µM FeCl_2_ (**b**), and 150 µM Fe_2_(SO_4_)_3_ (**c**) media, harvested 15 h after preparation of the media; and of precipitates adhering to sheaths in inoculated 300 µM FeSO_4_ (**d**), 300 µM FeCl_2_ (**e**), and 150 µM Fe_2_(SO_4_)_3_ (**f**) media, harvested after a three-day incubation. Insets in (**a**–**c**, **g**–**i**) are HRTEM images. The lack of lattice fringes indicates the amorphous state of these precipitates. (**B**) Distribution of inorganics in the precipitates illustrated in (**a**), determined by HAADF-STEM and EDX analyses. Note the detectable level of Fe, O, P, and Ca in all of these precipitates (**a**–**f** columns) irrespective of inoculation or uninoculation and Fe sources in the media. (**C**) Highly magnified TEM images of precipitates in uninoculated 300 µM FeSO_4_ medium (**a**) and highly magnified SE image acquired by STEM of those adhering to sheath surface in the inoculated medium (**b**). Note the precipitates entangled with numerous fine fibrils in (**b**). Highly magnified HAADF-STEM image of fibrillar cluster seen apart from the sheath (**c**).

Fe-associated precipitates formed in media containing Fe(II) or Fe(III) were analyzed by transmission electron microscopy (TEM). The precipitates in an uninoculated FeSO_4_ or FeCl_2_ medium were composed of nearly globular granules approximately 50 nm in diameter (Figure 4A a,b), while those in an uninoculated Fe_2_(SO_4_)_3_ medium were composed of large particles more than 3 µm in diameter (Figure 4A c). Nevertheless, the high-resolution TEM (HRTEM) images showed that the smaller granules and the larger particles had a similar amorphous structure with random atomic arrangement (Figure 4A a–c, inset). The subsequent scanning transmission electron microscopy (STEM)-EDX analysis detected Fe, P, O, and Ca in the precipitates and/or particles in all media (Figure 4B a–c, columns). All these results suggested that the basic texture of all precipitates have a similar amorphous structure and chemical composition in spite of their morphological differences.

A number of fine precipitates adhered to sheaths by day 3 in FeSO_4_ and in FeCl_2_ medium (Figure 4A g,h). The sheaths were almost entirely covered with large particles in the Fe_2_(SO_4_)_3_ medium (Figure 4A i). At high magnification, the morphology and amorphous structure of these precipitates and particles could be seen to be similar to those in the uninoculated media (Figure 4A d–f, inset). The EDX mapping images as well as the Mössbauer data demonstrated that the fine precipitates in FeSO_4_ and FeCl_2_ media and the large particles in Fe_2_(SO_4_)_3_ medium contained Fe(III), P, O, and Ca, irrespective of the presence (Figure 4B d–f, columns and Figure S2) or absence of the cells (Figure 4B a–c, columns and Figure S2). These results showed that the existing Fe-based oxides were formed by abiotic oxidation before adherence to the initial sheaths in media that contained Fe(II) or Fe(III) compounds.

Intriguingly, the precipitates that formed in 300 µM FeSO_4_ medium (Figure 4B a, C a) were entangled with fine fibrils (1–2 nm wide) of bacterial origin when the inoculated medium was incubated for three days (Figure 4B d, C b). Similar fine fibrils were scattered in places apart from the sheath surfaces (Figure 4C c). A similar phenomenon was also observed in the inoculated 300 µM FeCl_2_ medium (Figure 4B b,e).

### 3.6. Fe Deposition onto Sheaths Unrelated to Bacterial Cells

**Figure 5:**
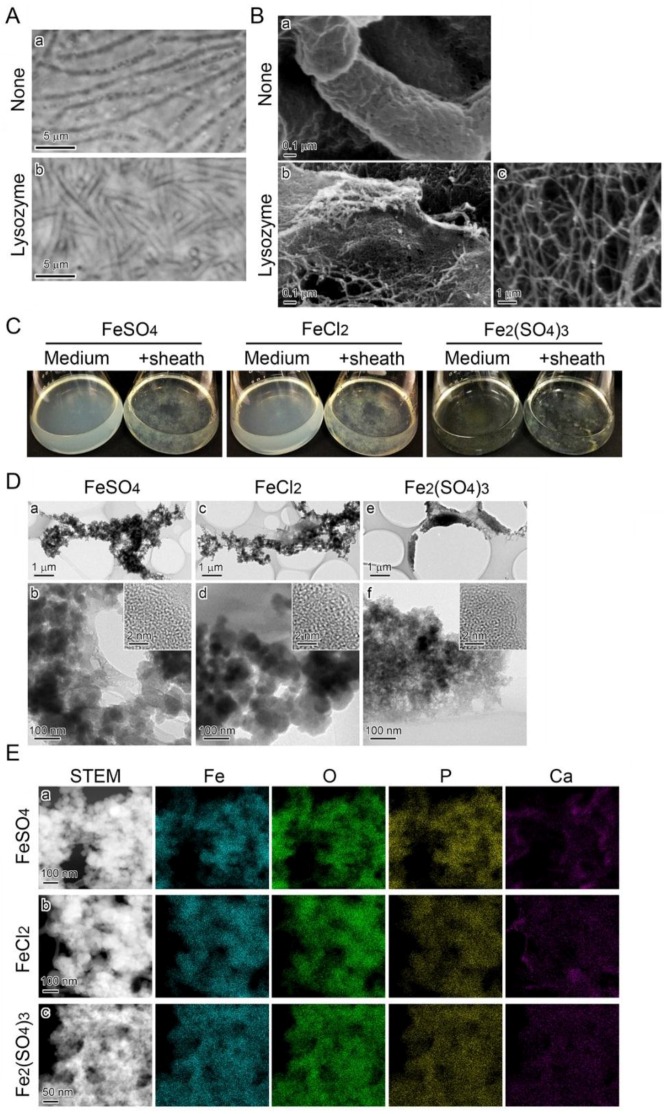
Deposition of precipitates onto SP-6 cell-free sheaths in media containing Fe(II) or Fe(III). (**A**) Phase contrast micrographs of sheaths enveloping bacterial cells before the lysozyme-EDTA-SDS treatment (**a**) and empty sheaths after the treatment (**b**). (**B**) SEM images of sheaths harvested from MSVP medium after a three-day incubation before (**a**) and after (**b**) the lysozyme-EDTA-SDS treatment. Note that nanoscaled fibrils released the sheath after the treatment (**b**). (**C**) Fluffy mass of complexes of medium precipitates and cell-free sheaths in 300 µM FeSO_4_ (left), 300 µM FeCl_2_ (middle), and 150 µM Fe_2_(SO_4_)_3_ (right) media compared with the respective clear, uninoculated medium (left of the flask pair). (**D**) TEM images of precipitates adhering to the sheath surfaces at low (**a**,**c**,**e**) and high magnifications (**b**,**d**,**f**), harvested from 300 µM FeSO_4_ (**a**,**b**), 300 µM FeCl_2_ (**c**,**d**), and 150 µM Fe_2_(SO_4_)_3_ (**e**,**f**) media after a two-day incubation. Insets in (**b**,**d**,**f**) are HRTEM images. The lack of lattice fringes in the images indicates the amorphous state of these precipitates. (**E**) Distribution of Fe, O, P, and Ca detected by EDX in complexes of the precipitates and sheath fibrils harvested from the respective media.

Sheaths produced in MSVP medium (Figure 5A a) were treated serially with lysozyme, EDTA, and SDS to lyse the enveloped cells. Phase contrast microscopy proved that all of the cells were lysed inside the sheath after this serial treatment (Figure 5A b). Before the treatment, the sheath surface was covered with aggregated fibrous material (Figure 5B a); after the treatment, the cell-free sheaths appeared to be sheet-like structures composed of aggregated nanoscaled fibrils (Figure 5B b). The mass of scattered nanoscaled fibrils was visible at high SEM magnification (Figure 5B c). When these cell-free sheaths were added to an FeSO_4_, FeCl_2_, or Fe_2_(SO_4_)_3_ medium, the precipitates disappeared from all media within two days; instead, fluffy sheath complexes that were mixed with the precipitates appeared on the flask bottom (Figure 5C). TEM revealed that the sheath fibrils enveloped the adhering, granular precipitates that had an amorphous structure (Figure 5D). A subsequent STEM-EDX detected Fe, O, P, and Ca in these adhering precipitates (Figure 5E). The morphology and components of these precipitates were consistent with those formed in the respective media and cell-containing sheaths, as described (Figure 4A).

## 4. Discussion

The present experiments demonstrate the occurrence of direct deposition of Fe-containing precipitates onto *Leptothrix* sheaths in media containing Fe salts. Abiotically formed Fe complexes became deposited onto sheaths, in particular, at the incipient stage of sheath formation and at high Fe concentrations in the medium. The addition of 300 µM FeSO_4_ or FeCl_2_ to MSVP-Fe medium caused prompt visible precipitation within 20 min (Figure 2A). Importantly, the precipitation-related turbidity gradually reduced as the cells multiplied (Figure 2A). The SEM images showed that single or aggregated fine precipitates adhered to the sheath surface in the inoculated media during a three-day incubation (Figure 1E d and Figure 2E b). The subsequent electron microscopic analyses revealed that these fine precipitates were composed of nearly globular granules (*ca*. 50 nm diameter) with an amorphous structure and EDX-detectable levels of Fe, P, O, and Ca, which were identical to the EDX analytical characters of the precipitates in the uninoculated media (Figure 4A,B), leading us to conclude that the Fe precipitates were directly deposited onto the sheath surfaces in a 300 µM Fe(II) medium.

In contrast, the surface of sheaths in the inoculated 10 µM FeSO_4_ or FeCl_2_ medium after a three-day incubation was smooth without any adhering precipitates (Figure 1E b and Figure 2E a). Intriguingly, the EDX analysis failed to detect any Fe deposition in these sheaths (Figure 1F a,b and Figure 2E a,b), suggesting that the degree of Fe deposition could depend on the duration of culture and/or the amount of Fe precipitates in the medium. To verify this assumption, we cultured the cells in 10 µM FeSO_4_ or FeCl_2_ medium by transferring the collected cells into the newly prepared respective 10 µM Fe(II) media at 2–3-day intervals for 10 days to recover the initial level of the Fe source. The SEM observation proved that a number of aggregated precipitates adhered to the surface of sheaths in these media, and in the EDX analysis, copious amounts of Fe and P were found in the entire sheath and in the adhering precipitates (Figure S3). The prompt decline in the Fe(II) concentration supplied to the medium (Figure 2B) and the supplemental Mössbauer results (Figure S2) indicated rapid abiotic oxidation of Fe(II) to Fe(III) in the medium, as supported by a report on rapid autoxidation of Fe in an aquatic system [14,22]. Therefore, most likely, in a liquid phase containing a certain level of Fe(II) such as 300 µM, rapid abiotic autoxidation forms enough Fe precipitates to cause Fe deposition on the sheath surfaces; this process is slower when the phase contains a low level of Fe(II) such as 10 µM. The slower Fe deposition at the low level of Fe(II) may reflect the possibility that the cells may be taking up Fe for growth.

Fluffy, brownish precipitates formed in media within 1 h of the addition of Fe(III) from 5 to 250 µM Fe_2_(SO_4_)_3_ (Figure 3G). These precipitates did not cause the visible turbidity typical of the fine precipitates in Fe(II)-containing media (Figure 2A,D). They formed a brownish mass together with sheaths on the flask bottom within 24 h of the addition of cells to these media (Figure 3G). The TEM and STEM analyses proved that the precipitates in the Fe(III)-containing medium were much larger than those in the Fe(II)-containing media, but both precipitates were very similar in their amorphous structure and inorganic components (Figure 4A a–c, B a–c). In addition, the precipitates in the Fe(III)-containing medium adhered to the sheath surface directly, similar to the case of the Fe(II)-containing media (Figure 4A f,i, B f). Because EDX-detectable levels of P and Ca, which were derived from the media, were found in the precipitates in the Fe-containing media (Figure 4B a–c), inorganic elements other than Fe could be involved in forming presumable hydroxides and/or oxyhydroxides; thus, they might not be a simple complex of Fe salts.

Results on the association of Fe complexes preformed in media with the sheath surfaces raised another query: Does the direct deposition of Fe precipitates onto sheaths require the presence of bacterial cells? To answer this question, cell-free sheaths were prepared by lysozyme-EDTA-SDS (LES) treatment (Figure 5A,B) and then treated with the Fe(II)- or Fe(III)-containing medium. SEM observations revealed that the basic skeleton of sheaths was composed of an assembly of nanoscaled fibrils (Figure 5B b,c). Incubation of the cell-free fibril assembly in the Fe(II)- and in the Fe(III)-containing media showed that apparently the Fe precipitates were abiotically deposited on the assembly surfaces (Figure 5D,E), independent of cell activity. Deposition was also similar on sheaths surrounding streptomycin-killed cells (Figure S4) and on protein-free sheaths (Figure S5) that were incubated in 300 µM FeSO_4_, 300 µM FeCl_2_, or 150 µM Fe_2_(SO_4_)_3_ media for two days. In the same manner as for the LES-treated sheaths, precipitates were deposited on the sheath surfaces, confirming that biological activity is not necessarily relevant to the disputed deposition.

Several earlier EDX- or EELS-mapping studies showed a nearly uniform distribution of inorganics in the *L. ochracea* sheaths that were harvested from natural environments [6] or from a relatively long-term (7–14 days) culture [12,20], giving the impression that active groups of organics in the bacterial exopolymer fibrils, which construct the basic frame of the sheaths, contribute to attracting and binding aqueous-phase inorganics [27]. Here, TEM and STEM images revealed that exopolymer fibrils extending from the sheath surfaces were entangled with Fe precipitates that adhered to the sheaths in 300 µM Fe(II) media (Figure 4C,B d,e), leading us to suspect that such an entangling net might account for the direct Fe deposition and ask whether the active groups of organics are involved in this interaction. We are now investigating what factor(s) and conditions trigger the abiotic, direct deposition of Fe and how the precipitates bind to exopolymer fibrils that extend from the sheaths.

The kinetics of abiotic Fe(II) oxidation was compared with oxidation in the presence of *L. cholodnii* Appels in a batch culture using a medium containing Fe salts under varied oxygen levels [18]. The results suggested that the competitive advantage of microbial iron oxidation in low-oxygen environments might be limited by the autocatalytic nature of Fe(III) oxidative precipitation, unless the accumulation of Fe(III) oxides was prevented, for example, through a close coupling of Fe(II) oxidation and Fe(III) reduction. Therefore, the possibility remains that the abiotic Fe(II) oxidation and its products may interfere with the biotic Fe(II) oxidation in medium.

There is an increasing body of circumstantial evidence that specific *Leptothrix* species (e.g., *L. ochracea*) are chemolithoautotrophic, capable of harnessing the electrons generated during biogenic oxidation of aqueous-phase Fe(II) to Fe(III) as a driving force for metabolism [14], suggesting that Fe encrustation in sheaths could be closely associated with bacterial metabolism. Similarly, [27,28] performed spectromicroscopic studies on microbially generated submicrometer-diameter iron oxyhydroxide filaments in a natural iron oxyhydroxide-encrusted biofilm inhabited by *Gallionella* and *Leptothrix* spp. and presented clear evidence that the polysaccharide strands that are excreted from the cells localized FeOOH, akagenite, precipitation close to the cell membrane to harness the proton gradient for energy generation. In addition, we have to consider the ecophysiology of Fe-oxidizing microbes such as coordination and interaction of inhabiting microbes for Fe oxidation and interference of extracellular polymers from a couple of inhabiting microbes with Fe oxide growth, as reported previously [28,29,30] Their reports evidently showed that in natural environments biogenicity and the microbial metabolism are more complex than in artificial culture conditions.

The present study fails to explain how attachment of Fe(III) particles onto sheaths in the medium relates to and interferes with abiotic precipitation, which was previously reported [27,28]. We certainly need to further investigate abiotic Fe oxidation to elucidate the Fe encrustation mechanism of sheaths in an artificial medium instead of natural environments. Nevertheless, here we would like to emphasize that we must be careful to interpret the experimental data in artificial conditions when consideration is linked to phenomena in natural environments.

## 5. Conclusions

The present results demonstrated that when *L. cholodnii* SP-6 cells were cultured in media amended with high Fe(II) concentrations, Fe(III) precipitates visibly formed immediately after addition of Fe(II) to the medium, suggesting prompt abiotic oxidation of Fe(II) to Fe(III) in the medium. Intriguingly, these precipitates were deposited onto the sheath surface surrounding bacterial cells as their population was actively growing. When Fe(III) was added to the medium, similar precipitates formed in the medium first and were then abiotically deposited onto the sheath surfaces. The precipitates in the Fe(II) medium were composed of assembled globular, amorphous particles (*ca.* 50 nm diameter), while those in the Fe(III) medium were composed of large, aggregated particles (≥ 3 µm diameter) with a similar amorphous structure. These precipitates also adhered to cell-free sheaths, leading us to conclude that direct abiotic deposition of Fe complexes onto the sheath surface occurs independently of cellular activity in liquid media containing Fe salts, although we need further analyses to determine how this deposition ties into the previously proposed mechanisms (oxidation enzyme- and/or active group of organic components-involved) of Fe encrustation of the *Leptothrix* sheaths.

The mechanisms of biological systems to produce extraordinary inorganic structures and morphologies are of great interest for engineering novel materials [28]. Already a variety of industrial functions such as lithium-ion battery anode material [31,32], catalyst enhancer [33,34,35], and porcelain pigment [36] have been discovered for the sheaths of *Leptothrix*. The present study suggests that the sheath materials may also be encrusted with metallic ions other than Fe; we thus hope to create novel metal-encrusted functional materials in the near future.

## Supplementary Materials

The following are available online at http://www.mdpi.com/2079-7737/5/2/26/s1, Figure S1: DIC and SEM micrographs of SP-6 sheaths in MSVP and MSVP-Fe after a three-day culture, Figure S2: Mössbauer spectra of precipitates formed in MSVP-Fe supplemented with 300 µM FeSO_4_, 300 µM FeCl_2_, or 150 µM Fe_2_(SO_4_)_3_, Figure S3: Deposition of Fe precipitates onto SP-6 sheaths by a 10-day culture in Fe(II)- or Fe(III)-containing medium, Figure S4: Deposition of precipitates onto sheaths surrounding streptomycin-killed cells in media containing Fe(II) or Fe(III), Figure S5: Deposition of precipitates onto protein-free sheaths in media containing Fe(II) or Fe(III).

## Abbreviations

The following abbreviations are used in this manuscript:

cfu	Colony-forming unit
DIC	Differential interference contrast microscope
PH	Phase-contrast microscope
SEM	Scanning electron microscopy
EDX	Energy dispersive X-ray spectroscopy
STEM	Scanning transmission electron microscopy
HAADF	High angle annular dark-field
HRETM	High-resolution transmission electron microscopy
