# Supplementary material for: Abiotic Deposition of Fe Complexes onto Leptothrix Sheaths
Source: Biology (Basel). 2016 Jun 3;5(2):26. doi: 10.3390/biology5020026 (PMC4929540; doi:10.3390/biology5020026)
Supplement: Supplementary file 1 [file biology-05-00026-s001.pdf]

Abiotic Deposition of Fe Complexes onto *Leptothrix* Sheaths

Tatsuki Kunoh, Hideki Hashimoto, Ian R. McFarlane, Naoaki Hayashi, Tomoko Suzuki, Eisuke Taketa, Katsunori, Tamura, Mikio Takano, Mohamed Y. El-Naggar, Hitoshi Kunoh and Jun Takada

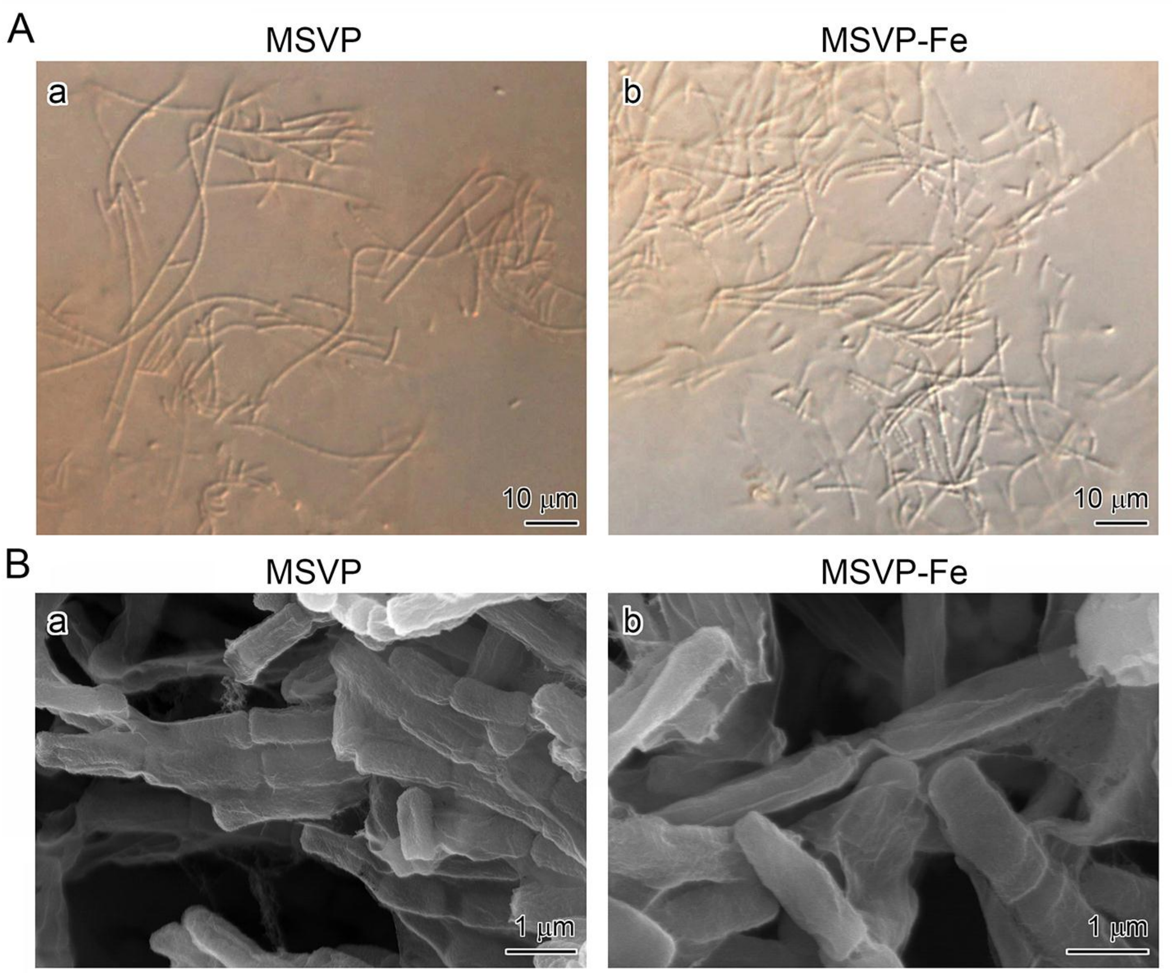

Figure S1. DIC (**A**) and SEM micrographs (**B**) of SP-6 sheaths in MSVP (**Aa, Ba**) and MSVP-Fe (**A b, B b**) after a three-day culture. The catenulate cells in MSVP were enveloped with extremely thin sheath walls. Thin sheaths (somehow shrunken) were also distinguished on the surface of catenulate cells in MSVP-Fe.

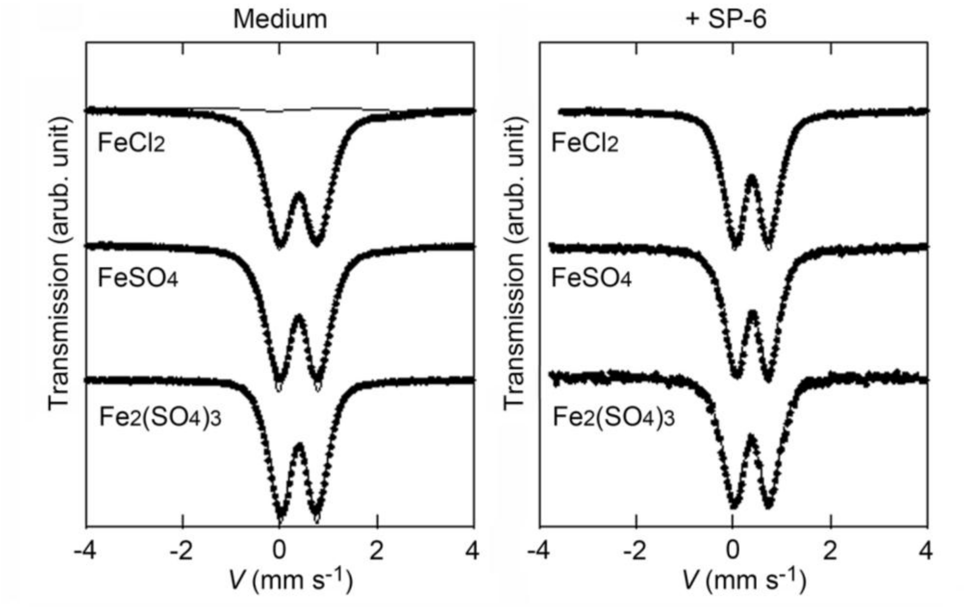

Medium	SP-6	Component	IS (mm s^{-1})	QS (mm s^{-1})	Width (mm s^{-1})	Area (%)
$FeCl_2$	−	Fe(III)	0.36	0.81	0.60	98
		Fe(II)	1.17	2.55	0.50	2
	+	Fe(III)	0.39	0.72	0.45	100
$FeSO_4$	−	Fe(III)	0.38	0.82	0.50	100
	+	Fe(III)	0.40	0.69	0.45	100
$Fe_2(SO_4)_3$	−	Fe(III)	0.39	0.77	0.46	100
	+	Fe(III)	0.38	0.75	0.48	100

Figure S2. Mössbauer spectra of precipitates formed in MSVP-Fe supplemented with 300 μM $FeSO_4$, 300 μM $FeCl_2$, or 150 μM $Fe_2(SO_4)_3$. Precipitates formed in these media (indicated as Medium) or incubated with SP-6 cells for three days (indicated as + SP-6) were harvested by centrifugation and freeze-dried. ^{57}Fe Mössbauer measurements were performed in transmission geometry using ^{57}Co/Rh as a radiation source and a-Fe as a control for velocity calibration and center shift. The collected spectra were computer-fitted using the Lorenzian function. The precipitates were composed of 100% Fe(III) in $FeSO_4$- and $Fe_2(SO_4)_3$-containing media, and 98% Fe(III) and 2% Fe(II) in an $FeCl_2$-containing medium, indicating that Fe(II) supplied to the medium is promptly oxidized to Fe(III).

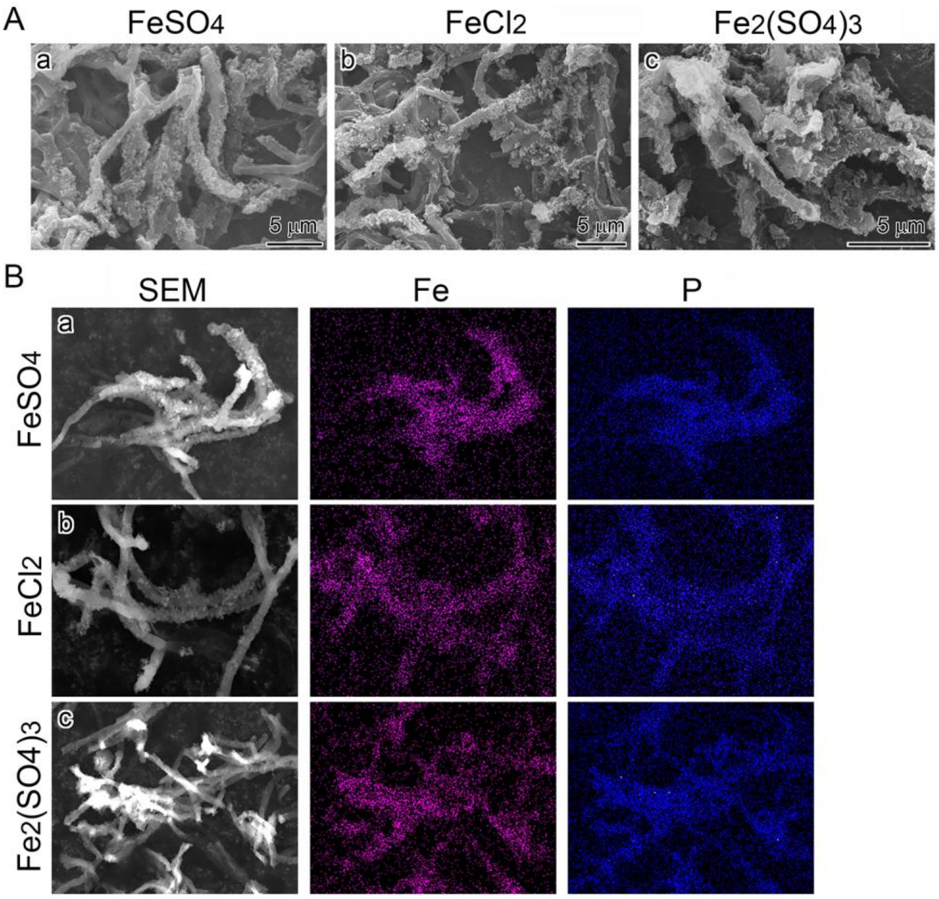

Figure S3. Deposition of Fe precipitates onto SP-6 sheaths by a 10-day culture in an Fe(II)- or Fe(III)-containing medium. SEM images (**A**) and EDX mapping images (**B**) of sheaths harvested from media containing $FeSO_4$ (**a**), $FeCl_2$ (**b**), and $Fe_2(SO_4)_3$ (**c**), with replacement of the newly prepared respective media at 2–3 day intervals. Note that the precipitates on the sheath surface and the entire sheath showed intense signals of Fe and P.

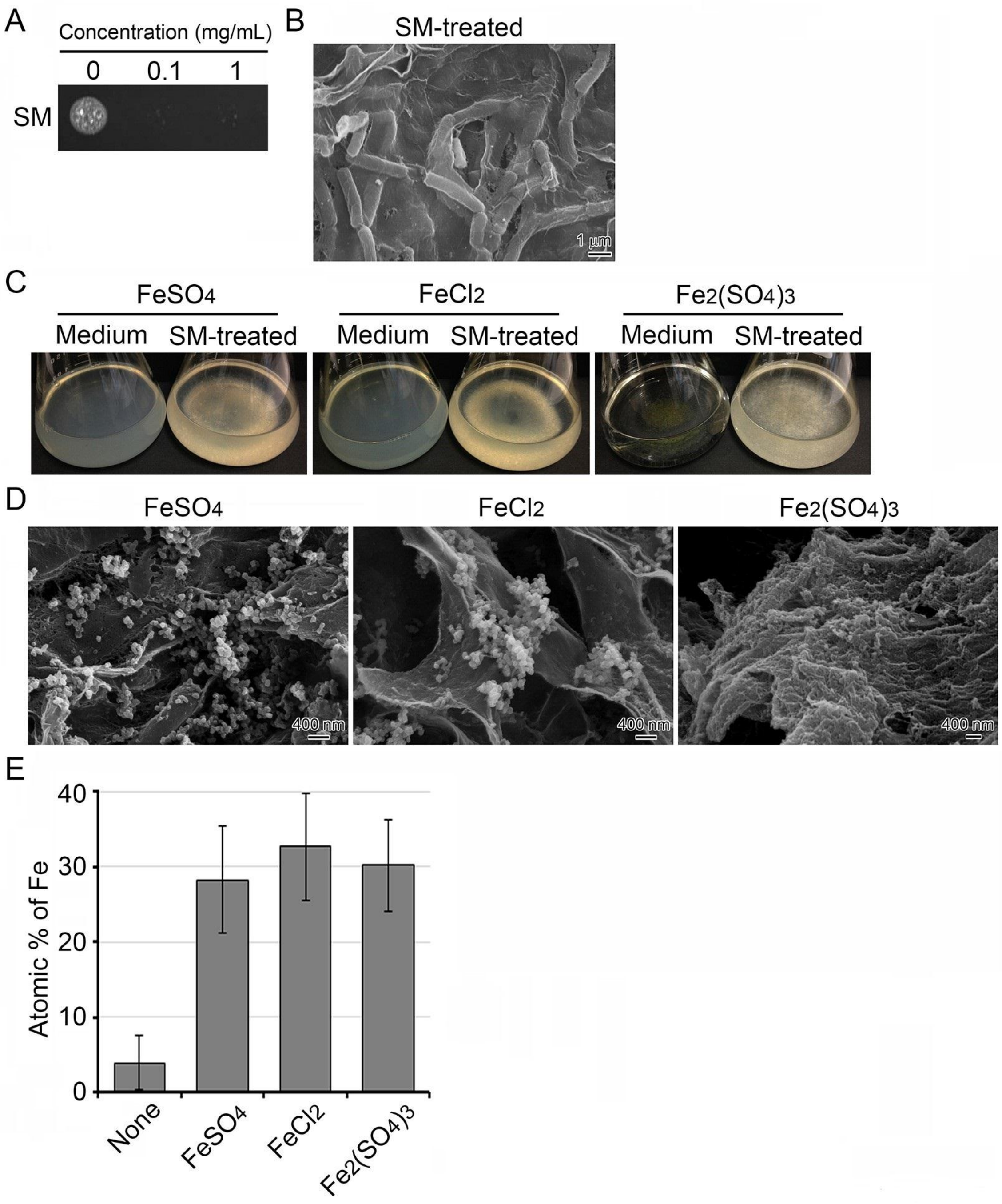

Figure S4. Deposition of precipitates onto sheaths surrounding streptomycin-killed cells in media containing Fe(II) or Fe(III). (**A**) SP-6 cells are sensitive to ≥0.1 mg/mL streptomycin (SM), as determined by a spot test on MSVP agar medium. (**B**) SEM image of SM-treated sheaths. (**C**) Fluffy complexes of medium precipitates and cell-killed sheaths in 300 μM $FeSO_4$ (left), 300 μM $FeCl_2$ (middle), and 150 μM $Fe_2(SO_4)_3$ (right) media. Each of the respective uninoculated, clear media is in the flask on the left. (**D**) SEM images of precipitates adhering to the aggregated sheath surfaces in 300 μM $FeSO_4$ (left), 300 μM $FeCl_2$ (middle), and 150 μM $Fe_2(SO_4)_3$ (right) media after a two-day incubation. (**E**) Atomic percentage of Fe in cell-killed sheaths harvested from $FeSO_4$, $FeCl_2$, or $Fe_2(SO_4)_3$ medium after a two-day incubation (determined by EDX). Atomic percentage of Fe in SM-treated sheaths without incubation in medium (none) was used as a control. Expressed as mean ± s. d. from N = 10 spots per sample.

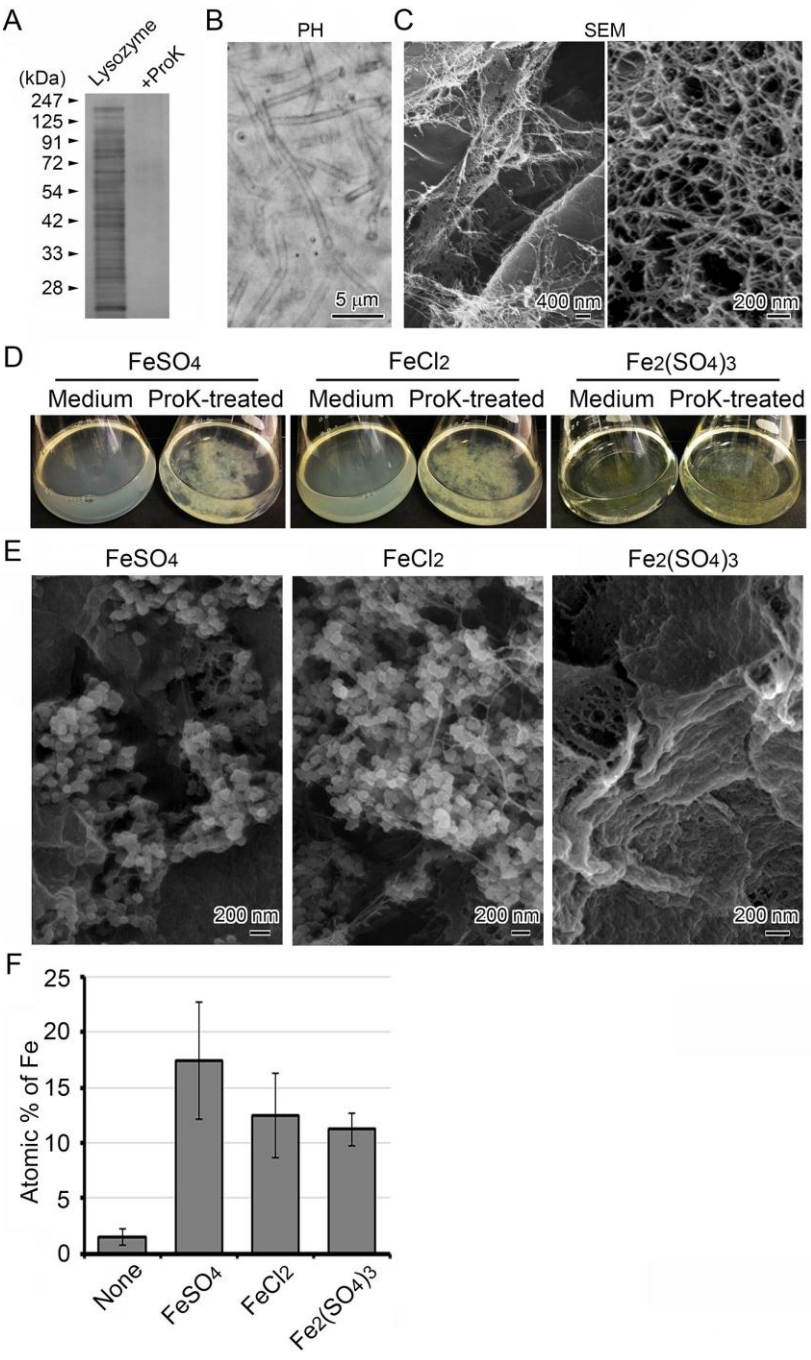

Figure S5. Deposition of precipitates onto protein-free sheaths in media containing Fe(II) or Fe(III). (**A**) Silver staining of SDS-PAGE gel loaded with sheath fractions after lysozyme-EDTA-SDS (left lane) or lysozyme-EDTA-SDS/proteinase K treatment (right lane). (**B**,**C**) Phase contrast micrograph (**B**) and SEM images (**C**) of empty sheaths after lysozyme-EDTA-SDS/proteinase K treatment. (**D**) Fluffy complexes of medium precipitates and protein-free sheaths in 300 μM $FeSO_4$ (left), 300 μM $FeCl_2$ (middle), and 150 μM $Fe_2(SO_4)_3$ (right) media. Each of the respective uninoculated clear media is in the flask on the left. SEM images of precipitates adhering to the aggregated sheath surfaces in 300 μM $FeSO_4$ (left), 300 μM $FeCl_2$ (middle), and 150 μM $Fe_2(SO_4)_3$ (right) media after a two-day incubation. (**E**) SEM images of precipitates adhering to the aggregated sheath surfaces in 300 μM $FeSO_4$ (left), 300 μM $FeCl_2$ (middle), and 150 μM $Fe_2(SO_4)_3$ (right) media after a two-day incubation. (**F**) Atomic percentage of Fe in protein-free sheaths harvested from $FeSO_4$, $FeCl_2$, or $Fe_2(SO_4)_3$ media after a two-day incubation (determined by EDX). Atomic percentage of Fe in Lysozyme-EDTA-SDS/Proteinase K-treated sheaths immediately after preparation (without incubation in medium [none]) was used as a control. Expressed as mean ± s. d. from $N = 10$ spots per sample.
